# Changes in the Leptin to Adiponectin Ratio Are Proportional to Weight Loss After Meal Replacement in Adults With Severe Obesity

**DOI:** 10.3389/fnut.2022.845574

**Published:** 2022-05-18

**Authors:** Mohammed Faraz Rafey, Razk Abdalgwad, Paula Mary O'Shea, Siobhan Foy, Brid Claffey, Colin Davenport, Derek Timothy O'Keeffe, Francis Martin Finucane

**Affiliations:** ^1^Bariatric Medicine Service, Centre for Diabetes, Endocrinology and Metabolism, Galway University Hospitals, Galway, Ireland; ^2^HRB Clinical Research Facility, National University of Ireland Galway, Galway, Ireland; ^3^Department of Medicine, National University of Ireland Galway, Galway, Ireland; ^4^Department of Clinical Biochemistry, Galway University Hospitals, Galway, Ireland

**Keywords:** meal replacement, hypocaloric diet, insulin resistance, leptin, adiponectin, leptin: adiponectin ratio, severe obesity, milk

## Abstract

Hypocaloric diets are known to induce changes in adipokine secretion, but the influence of a low energy liquid diet (LELD) on the leptin: adiponectin ratio (LAR), a measure of insulin resistance and cardiovascular risk, has not previously been investigated in patients with severe obesity. We conducted a prospective, single-center cohort study of adults with severe obesity (defined as body mass index (BMI) ≥40 kgm^−2^, or ≥35 kgm^−2^ with co-morbidities) who completed a 24-week milk-based LELD. We measured leptin, adiponectin and LAR at the start and on completion of the programme. Of 120 patients who started, 52 (43.3 %) completed the programme. Their mean age was 50.3 ± 11.2 (range 18–74) years, 29 (55.8 %) were female and 20 (38.5 %) had type 2 diabetes mellitus (T2DM). Weight decreased from 148.2 ± 39.6 to 125.4 ± 34.8 kg and BMI decreased from 52.4 ± 11.1 to 44.3 ± 9.8 kgm^−2^, respectively (all *p* < 0.001). In patients with T2DM, HbA1c decreased from 60.0 ± 17.4 to 47.5 ± 15.5 mmol/mol (*p* < 0.001). Leptin decreased (from 87.2 [48.6, 132.7] to 39.1 [21.0, 76.4] ng/ml) and adiponectin increased (from 5.6 [4.5, 7.5] to 7.1 [5.5, 8.5] μg/ml), with a reduction in LAR from 15 [8.4, 22.4] to 5.7 [3.0, 9.1] ng/μg (all *p* < 0.001), indicating decreased insulin resistance. The percentage weight lost was associated with the percentage reduction in LAR (ß = 2.9 [1.7, 4.1], *p* < 0.001) and this association was stronger in patients with T2DM. Patients with severe obesity who completed a milk-based LELD had a substantial reduction in LAR, consistent with decreased insulin resistance and cardiovascular risk, proportional to weight loss.

## Introduction

Obesity is an important contributor to type 2 diabetes (T2DM) risk, primarily through its adverse effects on insulin sensitivity (1). These effects are associated with a disruption of normal adipose tissue physiology, hyperplasia and hypertrophy of adipocytes leading to a pro-oxidative and pro-inflammatory thrombotic state, along with altered production of the adipokines leptin and adiponectin (2). Both of these hormones are predominantly secreted by adipocytes (with some gastric production in the case of leptin) (3), and they are important regulators of metabolic homeostasis that become abnormal in the disease of obesity. Leptin acts on the hypothalamus to regulate food intake and energy expenditure (4) and is known to be elevated in obesity (5). Conversely, adiponectin increases tissue fat oxidation, reduces circulating free fatty acids, and is lower in individuals with obesity (6). The importance of these hormones to metabolism is highlighted by the observation that the ratio of leptin to adiponectin (LAR) is a robust measure of whole-body insulin sensitivity, validated in large epidemiological studies (7, 8). Gold standard measures of insulin sensitivity such as the hyperinsulinaemic-euglycaemic clamp can be relatively time consuming and expensive to perform (9) and this has limited their use in studies of the precise mechanisms of improvements in glycemic control with meal replacement programmes (10–13).

To date, several studies have described changes in leptin and adiponectin levels in response to weight-loss interventions. A recent systematic review has shown that bariatric surgery leads to reduced leptin and increased adiponectin in adults with severe obesity (14). Changes in these hormones have been shown to predict diabetes remission after surgery (15). Similar reductions in leptin and increases in adiponectin have been described in adolescents (16) and adults (17) undergoing lifestyle modification for weight loss. However, these studies have not assessed changes in LAR specifically as a measure of insulin sensitivity. We sought to determine the influence of completion of a calorie-restricted low energy liquid diet (LELD) on leptin, adiponectin and in particular, the LAR in a cohort of patients with severe obesity (with and without T2DM). We also sought to determine whether changes in LAR are associated with either the magnitude of weight loss achieved or improvements in glycemic control, in order to better understand the mechanisms by which glycaemic control improves with meal replacement programmes.

## Methods

We conducted a single-center, prospective observational cohort study of patients with severe obesity who undertook and completed a milk-based LELD. Thus, our study describes baseline and follow-up measures only in programme completers, with no follow-up measures for those who dropped out and with no control group for comparison. The study was conducted in accordance with the STROBE (Strengthening the Reporting of OBservational studies in Epidemiology) guidelines (18). Patients who were enrolled in our out-patient, milk-based LELD between November 2017 and May 2019 at the Bariatric Medicine Service in Galway University Hospital were invited to participate in this study. The study was approved by the Galway University Hospitals' Central Research Ethics Committee in December 2017 (ref CA 1802).

### Inclusion and Exclusion Criteria

Male and female patients aged 18 years or older referred to the bariatric service for assessment of severe obesity were eligible for inclusion. Our clinical practice is to define severe obesity as a BMI ≥ 40 kg m^−2^ (or ≥35 kg m^−2^ with co-morbidities such as type 2 diabetes or obstructive sleep apnea syndrome). Patients must have been willing to attend all of the 14 scheduled study visits. Female patients of childbearing potential who were pregnant, breastfeeding, or intended to become pregnant or were not using adequate contraceptive methods were not considered eligible for the programme. Those with a recent myocardial infarction (within 6 months), untreated arrhythmia, untreated left ventricular failure, recent cholelithiasis (within the past year), hepatic or renal dysfunction, type 1 diabetes, untreated major psychiatric disorders, eating disorders, cancer, previous bariatric surgery, a BMI <35 kg m^−2^ or those deemed unlikely to attend for the full programme (e.g., frequent prior clinic non-attendance) were excluded from the programme. We also excluded patients with an acute inflammatory disorder as this may have influenced adipokine levels. All participants provided written informed consent. They attended the bariatric clinic every 2 weeks for 24 weeks, with 14 visits in total. Leptin and adiponectin levels were measured at two timepoints only – week zero and week 24.

### Measurements

At each visit, participants met with the nurse, dietitian and physician. Weight was measured on a Tanita^®^ scale and height with a Seca^®^ wall-mounted stadiometer. Fasting venous whole blood (20ml) was collected into Becton Dickinson BD Vacutainer^®^ plain plastic containers (for leptin and adiponectin) and potassium ethylenediaminetetraacetic acid (EDTA) plasma [for glycosylated hemoglobin (HbA1c)], measured using High-Performance Liquid Chromatography (HPLC) on the Menarini^®^ HA8160 analyser. Samples for leptin and adiponectin were transferred to the laboratory within 20 min of collection where they were centrifuged at +4°C at 3,600 rpm for 10 min, following which serum was transferred to polystyrene secondary tubes (Roche), sealed and stored at −20°C until subsequent batch analysis using separate two-site micro titer plate-based DELFIA assays (manufactured by R&D Systems Europe, Abingdon UK). The adiponectin assay measured “total” adiponectin with inter-assay imprecision of 5.4% at 3.6 μg/ml, 5.2% at 9.2 μg/ml and 5.8% at 15.5 μg/ml, as previously described (19). For leptin, the between batch imprecision at concentrations of 2.7 ng/ml, 14.9 ng/ml and 54.9 ng/ml was 7.1, 3.9 and 5,7% respectively.

### Intervention

The milk-based LELD consisted of three continuous 8-week phases, each with fortnightly visits to the bariatric medicine clinic. During the first (weight loss) phase from weeks one to eight inclusive, an exclusively milk-based liquid diet was prescribed, consisting of approximately 2.5 liters per day of semi-skimmed milk divided in seven portions throughout the day in equal doses, with additional sodium replacement, vitamin, mineral, and fiber supplementation, equivalent to approximately 1,200 kcal/day, or 130 g of carbohydrates and 40 g of fat intake per day. The precise caloric content and volume of milk was determined by each participant's baseline body weight, from which their total daily protein requirements (in grams per day) were calculated using 0.17g N_2_/ kg × 6.25. For patients with a BMI <50 kg m^−2^, we replaced 75% of these daily protein requirements whereas for those with a BMI was ≥50 kg m^−2^, 65% of total daily protein requirements were replaced. This was equivalent to approximately 130g of protein per day, as we have described previously (20). Throughout this first 8-week phase of the programme, renal and liver profiles were assessed every 8 weeks and the patient was seen by the clinical team at each visit. During the second phase (weight stabilization) from weeks 9 to 16 inclusive, there was a gradual re-introduction of low-calorie meals from a set menu over 8 weeks, under the supervision of the bariatric dietitian with fortnightly visits continuing. During the third phase (weight maintenance) from weeks 17 to 24 inclusive, the milk component of the diet was stopped completely and a fully solid isocaloric diet was restarted, based on individualized meal plans, again under the supervision of the bariatric dietitian.

### Statistical Methods

All statistical analyses were conducted with SPSS^®^ version 26. The Shapiro-Wilk test was used to determine normality in baseline measures. Changes in anthropometric and metabolic variables between baseline and completion of the programme were assessed using the student's paired *t*-test for normally distributed data. Excess body weight percentage was calculated using the formula {([BMI]/25) × 100}−100. Where variables were not normally distributed, the Wilcoxon signed-rank test was used. Categorical variables were compared using the Chi-Square test. Correlation between the degree of weight loss and changes in leptin, adiponectin, and LAR was determined using the Spearman coefficient. Associations between percentage change in weight (as the independent or predictor variable) and percentage change in leptin, adiponectin, and LAR (as the dependent or outcome variables) were determined using linear regression.

## Results

Of the 120 patients who started our milk-based LELD between November 2017 and May 2019, 52 (43.3 %) patients agreed to participate in this study, completed all 24 weeks of the programme and were included in the study analysis. Of these intervention completers, 29 (55.8%) were female and the mean age was 50.3 ± 11.2 (range 18–74) years. The mean height was 1.67 ± 0.09 m. Most patients had “White Irish” self-reported ethnicity (n = 44/ 84.6%), while three (5.76%) reported “Polish White” and one patient each described being of “Irish Traveler”, “German Jewish”, “German White”, “Italian White” and “Pakistani Asian” ethnicity. Obesity-related co-morbidities were highly prevalent, with 20 (38.5%) patients having T2DM, 37 (71.2%) being treated for hypertension, and 19 (36.5%) taking lipid-lowering therapy. Eight patients had asthma and two had rheumatoid arthritis but these were well controlled without clinical evidence of acute inflammation at any stage before or during the intervention. Changes in anthropometric and metabolic characteristics are shown in Table 1. There was a 22.8 ± 9.7 kg reduction in weight, with a reduction in BMI of 8.0 ± 3.2 kg m^−2^ and an absolute reduction in excess body weight percentage (EBW%) of 32.1 ± 12.9 % (all *p* < 0.001), equivalent to a percentage total weight loss of 15.3 ± 6.0 %. We found that 50 of the 52 patients (96.2%) achieved a total weight loss of 10% or more after 24 weeks.

**Table 1:** Changes in anthropometric variables, leptin, adiponectin and the LAR after completion of the milk-based LELD.

**Variable**	**Pre**	**Post**	***P* value**
Weight (kg)	148.2 ± 39.6	125.4 ± 34.8	<0.001
BMI (kg/ m-2)	52.4 ± 11.1	44.3 ± 9.8	<0.001
EBW (%)^†^	103.1 [78.7, 138.0]	73 [47.2, 96.0]	<0.001
Systolic Blood Pressure (mmhg)	126.5 ± 14.3	122.7 ± 13.7	0.048
Diastolic Blood Pressure (mmhg)	68.9 ± 11.3	69.2 ± 10.3	0.843
HbA1C (mmol/mol)**	38.2 ± 3.5	35.0 ± 3.1	<0.001
HbA1C (mmol/mol)*	60 ± 17.4	47.5 ± 15.5	0.001
Leptin (ng/ml)^†^	87.2 [48.6, 132.7]	39.1 [21.0, 76.4]	<0.001
Leptin (ng/ml)^†^**	92.8 [60.3,153.7]	42.8 [27.3,77.9]	<0.001
Leptin (ng/ml)^†^*	74.9 [35.9,111.7]	44.5 [17.6,60.8]	0.001
Adiponectin (μg/ml)^†^	5.6 [4.5, 7.5]	7.1 [5.5, 8.5]	<0.001
Adiponectin (μg/ml)^†^**	6.2 [5.0,8.6]	8.1 [6.5,10.6]	<0.001
Adiponectin (μg/ml)^†^*	4.8 [3.1,3.6]	6.0 [4.2,7.6]	0.004
LAR (ng/μg)^†^	15.0 [8.4, 22.4]	5.7 [3.0, 9.1]	<0.001
LAR (ng/μg)^†^**	11.7 [7.8,25.6]	5.6 [3.0,8.7]	<0.001
LAR (ng/μg)^†^*	16.0 [12.1, 21.2]	6.0 [3.0, 10.1]	0.002

All variables are presented as means ± standard deviation or for non-normally distributed data, medians and [interquartile ranges]. ^**^Denotes subgroup of patients without a history of type 2 diabetes. ^*^Denotes subgroup of patients with type 2 diabetes. †Denotes variables that are not normally distributed.

We found a significant reduction in HbA1c in the group overall, both in patients with T2DM and those without diabetes, as shown. Of those patients with T2DM, 14 out of 20 (70 %) had a Hba1C less than 48 mmol/mol at the end of the programme, and 8 out of 20 (40%) had 1 or more anti-hyperglycaemic drugs stopped during the programme (*p* = 0.007). Six (30%) patients were taking insulin prior to the start of the programme, while four (20%) remained on it at completion (*p* < 0.001). Six (30%) patients taking a sodium-glucose linked transported 2 (SGLT2) inhibitor remained on this drug at the end of the programme. Seventeen (85%) patients who were on metformin remained on it throughout the programme. Nine (45%) patients were on glucagon-like peptide 1 (GLP1) agonist therapy prior to the start of the programme and 7 (35%) remained on it at completion (*p* < 0.001). Of the two (10%) patients who were on sulphonylurea therapy at baseline, both discontinued it by completion (*p* < 0.001) and the one (5%) patient on a dipeptidyl peptidase 4 (DPP4)-inhibitor stopped this at the start of the programme. In total, 37 (71.1%) patients were on one or more anti-hypertensive agents at baseline, and 18 (34.6%) remained on this treatment at completion (*p* = 0.002), whereas 19 (36.5%) were on lipid lowering therapy and all remained on those drugs at completion. Overall, 21(40.3%) of patients had either one or more of their medications stopped during the programme (*p* = 0.006).

There were reductions in leptin and increases in adiponectin, with a significant reduction in LAR, as shown in Table 2. The magnitude of the changes in these variables was similar in the subgroup with T2DM to the overall group. When the 10 patients with asthma or rheumatoid arthritis were excluded, the results were also similar (data not shown). In linear regression analyses, with percentage weight loss as the independent or exposure variable, the magnitude of the percentage changes in leptin and LAR was strongly associated with weight loss, but the magnitude of the percentage change in adiponectin was only associated with weight loss in patients with diabetes, not in patients without diabetes, as shown in Table 2. So, for example, in patients with type 2 diabetes, for every percentage reduction in body weight, there was a 4.4% decrease in leptin, a 3.9% increase in adiponectin, and a 5.9% reduction in LAR. These results were similar after adjusting for age, sex, and baseline BMI (see Supplementary Table 1). We then sought to determine if there was any association between the percentage change in LAR as the independent variable and the percentage change in HbA1c as the dependent variable. We found no association between the two in patients with T2DM (ß = −0.57 [−1.6, 0.5], *p* = 0.285) or in patients without diabetes (ß = −0.75 [−2.0, 0.4], *p* = 0.224).

**Table 2:** Associations between percentage change in weight and percentage change in leptin, adiponectin and the LAR after completion of the milk-based LELD.

	**β**	**95% CI**	**P**
**Entire group (*****n** **=*** **52):**			
Δ Leptin%	2.9	[2.0,3.8]	<0.001
Δ Adiponectin %	0.4	[−4.3,5.1]	0.537
Δ LAR %	2.9	[1.7,4.1]	<0.001
**T2DM patients (*****n** **=*** **20):**			
Δ Leptin%	4.4	[2.0,6.8]	0.001
Δ Adiponectin %	−3.9	[−7.4, −0.5]	0.02
Δ LAR %	5.9	[2.6,9.1]	0.001
**Patients without DM (*****n** **=*** **32):**			
Δ Leptin%	2.4	[1.6,3.2]	<0.001
Δ Adiponectin %	2.1	[−4.9,9.2]	0.537
Δ LAR %	1.8	[1.0,2.7]	<0.001

*β denotes the beta coefficient and [confidence interval] for the estimate of the strength of the association between percentage weight change as the independent variable and percentage changes in leptin, adiponectin and LAR as the dependent variables, in unadjusted linear regression analyses. Results were similar after adjusting for age, sex and baseline BMI (see Supplementary Table 1)*.

**Figure 1:**
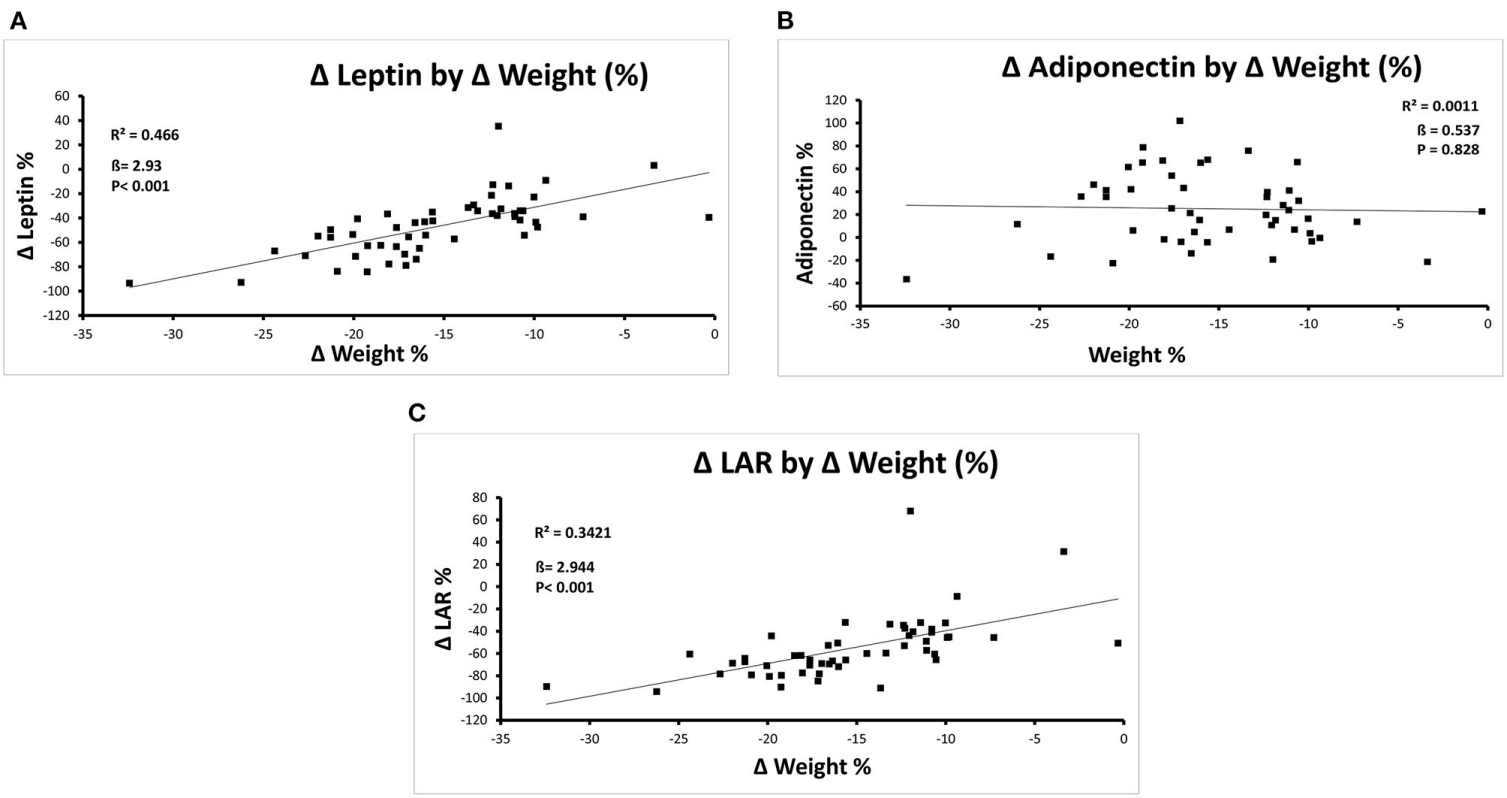
**(A–C)** Correlations between percentage change in weight and percentage change in leptin **(A)**, Adiponectin **(B)**, and the LAR **(C)** after Completion of the Milk-Based LELD.

There were significant correlations between percentage weight loss and percentage change in leptin and LAR, but not adiponectin, in the group overall, as shown in Figures 1A–C, such that 47% of the relative change in leptin and 34% of the relative change in LAR was accounted for by the relative change in weight. In the subgroup of patients with T2DM (Supplementary Figures 1a–c), results were similar, but there was also a correlation between weight change and change in adiponectin levels.

## Discussion

We have shown that in a cohort of adults with severe obesity who completed a 24-week milk-based LELD, there were substantial increases in adiponectin and reductions in leptin and LAR, consistent with increased insulin sensitivity. While a number of studies have described reductions in leptin and increases in adiponectin after dietary interventions (16, 17) and after bariatric surgery (21, 22), to date, none has considered changes in LAR per se. In a cohort of patients with T2DM undergoing bypass bariatric surgery, investigators described an increase in an “inverted” ratio of adiponectin: leptin, with broadly similar effects on both hormones to those that we have described here (23). Ours is the first study to consider these adipokines as a ratio in patients undergoing a meal-replacement based hypocaloric dietary intervention. We found that the reduction in LAR arose primarily from a reduction in leptin rather than an increase in adiponectin and that the change in LAR was proportional to weight loss, again consistent with findings described after gastric bypass (23). The magnitude of the changes we observed in leptin and adiponectin concentrations are broadly consistent with other studies, such as with commercial meal replacement programmes (24).

The magnitude of weight loss in our cohort is consistent with descriptions of other meal replacement programmes and LELD's (12, 25, 26). Of note, however, is our relatively short duration of follow-up and our inclusion only of patients who completed the dietary intervention in full. While some meal replacement programmes have reported reasonable weight loss maintenance at one- and three- years (27, 28), others have described substantial weight regain over time that limits longer-term efficacy of these interventions (29, 30). In fact, we have also recently described significant weight regain in patients we have followed for up to 4 years after completion of the milk-based LELD in our center (31). We would emphasize that this study is in no way trying to describe the efficacy or effectiveness of the milk-based LELD. Rather, our hypothesis relates to the relationship between weight loss and mechanistic improvements in metabolism. Given our recent observations of long term weight regain after the intervention (31), it is unlikely that any improvements in insulin sensitivity will be fully maintained over time.

We were surprised to find no association between the magnitude of the change in LAR and the change in HbA1c, given previous observations that in patients undergoing bariatric surgery, the magnitude of changes in adipokines predicted diabetes remission (15, 32). Our results may be due to inadequate statistical power, but we are inclined to reject this as the only reason we did not find an association. It may be that the improvement in HbA1c in our cohort arose from a mechanism that occurs independently of a change in adipocyte function. Improvements in glucose metabolism with dietary restriction and weight loss are known to arise from reduced insulin resistance but also enhanced beta-cell insulin secretion (33). In particular, changes in liver fat content and hepatic insulin resistance that arise from weight loss (34) are intricately linked with improvements in beta-cell function (35). Hypocaloric meal replacement in patients with T2DM has been shown to decrease ectopic fat in the liver and pancreas, but a critically important factor necessary for the remission of diabetes is the concomitant restoration of beta-cell function (36). Thus, it may be that the improvements we observed in glycaemic control in our patients arose from reduced pancreatic ectopic fat and enhanced beta-cell function. In retrospect, it is a limitation that we have not measured these specifically, especially given the growing realization that “adipose tissue” and “whole body” insulin resistance are distinct pathophysiological entities (37). Future studies could address some of these limitations by quantifying ectopic fat in specific organs, by measuring indices of beta-cell function, by including a control group, and by following patients up for a longer period of time. Specifically, it would be useful to incorporate measures of insulin resistance that are derived from fasting or post-glucose load glucose and insulin values (38, 39) rather than relying exclusively on adipokine values.

We did not measure or take account of physical activity or fitness, which is a limitation of our study, as the influence of exercise on leptin and adiponectin is well established: A meta-analysis of exercise interventions in children with obesity (40) and others in adults with pre-diabetes (41) and obesity (42) have described similar effects on adipokines as we have found here, but of lower magnitude. By quantifying physical activity and fitness, future studies could more robustly determine the mechanistic basis for improvements in metabolic health with dietary and physical activity components of lifestyle modification.

Another limitation of our study is its relatively modest size, though the large changes induced by the intervention allow a much smaller population with which to demonstrate an effect than many other weight loss interventions such as drugs (43) or lifestyle interventions (44). Our study population was also a relatively heterogeneous convenience sample, determined by the number of patients recruited to the milk-based meal replacement programme at our institution over the study period. Nonetheless, our findings around the correlation between weight loss and reduced LAR are statistically robust. As noted above, there were changes in diabetes medication usage within the cohort that are unavoidable in an observational study such as this, which might have influenced our results with regards to the association between LAR, weight loss, and changes in HbA1c. Metformin is known to increase insulin sensitivity (45) and reduce circulating levels of leptin (46), but there was no change in its usage in the study. SGLT2 inhibitors decrease leptin and increase adiponectin (47), but their use was also unchanged. GLP1 agonist therapy has previously been reported to attenuate the reduction in circulating levels of leptin after weight loss (48), and DPP4 inhibitors decrease leptin and increase adiponectin (49), so the reductions we observed in the use of these drugs would have led to an attenuation rather than an exaggeration of the change in LAR with weight loss. Angiotensin-converting enzyme (ACE) inhibitors increase adiponectin (50), so we think their cessation would have attenuated rather than enhanced the observed reduction in LAR.

Notwithstanding the metabolic improvements seen in patients completing the intervention, we must emphasize again that these findings offer mechanistic insights rather than any clinical evidence that LELDs are effective or beneficial to patients. As with similar studies of meal-replacement programmes, our attrition rate was very high, with more than half of patients dropping out (12, 26). Side effects of hypocaloric meal replacement programmes are common and include constipation, dizziness, alopecia, nausea, headache, diarrhea, abdominal pain, and cholelithiasis (51). Arguably our most important limitation is the lack of follow-up data for the patients who started the intervention but dropped out. Though unlikely, it is possible that some of those who dropped out may have had significant weight regain or serious adverse events that precluded continued participation, which we would not be aware of, which is why we have avoided any attempt to compare data in completers and non-completers using last- or mean- observation carried forward analyses.

## Conclusion

Patients with severe obesity who completed a milk-based LELD had significant short-term increases in adiponectin and reductions in leptin and the LAR, consistent with decreased insulin resistance, which was proportional to the amount of weight lost. Whether milk-based LELDs influence other indices of insulin resistance, are safe, effective or have sustainable benefits for metabolic health requires further studies.

## Data Availability Statement

The raw data supporting the conclusions of this article will be made available by the authors, without undue reservation.

## Ethics Statement

The studies involving human participants were reviewed and approved by Galway University Hospitals' Central Research Ethics Committee in December 2017 (ref CA 1802). The patients/participants provided their written informed consent to participate in this study.

## Author Contributions

MR contributed to study design, patient recruitment, delivery of the clinical intervention, data collection, conducted data analysis, and prepared the manuscript. RA contributed to patient recruitment, conducted data analysis, and helped in the preparation of manuscript. SF and BC contributed to patient recruitment and delivery of clinical intervention. PO'S processed biochemistry samples, collated metabolic data, and revised the manuscript. CD and DO'K contributed to the data analysis and revised the manuscript. FF conceptualised the study design, led the clinical intervention and supervised the data collection, drafting of the manuscript, and the data analysis. All authors have given final approval of the version to be published and agree to be accountable for all aspects of the work.

## Funding

This work was funded by a project grant from Healthy Ireland, a Government of Ireland initiative. FF was funded by a Clinical Research Career Development Award from the Saolta Hospital Group.

## Conflict of Interest

CD has either received honoraria or travel grants or has served on advisory boards for Novo Nordisk, Eli Lilly, Ethicon, Sanofi-Aventis, Astra Zeneca, Merck-Serono, Boehringer Ingelheim, Janssen and Novartis. DO'K has either received honoraria or travel grants or has served on advisory boards for Novo Nordisk, Eli Lilly, Ethicon, Pfizer Inc., Sanofi-Aventis, Astra Zeneca, Merck-Serono, Boehringer Ingelheim, Janssen and Novartis. The remaining authors declare that the research was conducted in the absence of any commercial or financial relationships that could be construed as a potential conflict of interest.

## Publisher's Note

All claims expressed in this article are solely those of the authors and do not necessarily represent those of their affiliated organizations, or those of the publisher, the editors and the reviewers. Any product that may be evaluated in this article, or claim that may be made by its manufacturer, is not guaranteed or endorsed by the publisher.
