# Supplementary material for: Changes in the Leptin to Adiponectin Ratio Are Proportional to Weight Loss After Meal Replacement in Adults With Severe Obesity
Source: Front Nutr. 2022 May 18;9:845574. doi: 10.3389/fnut.2022.845574 (PMC9158748; doi:10.3389/fnut.2022.845574)
Supplement: Supplementary file 1 [file Table_1.docx]

|  | **β** | **95% CI** | **P** |
| --- | --- | --- | --- |
| **Entire group (n=52):** |  |  |  |
| Δ Leptin% | 2.9 | [2.1, 3.8] | <0.001 |
| Δ Adiponectin % | 0.29 | [-4.5, 5.1] | 0.904 |
| Δ LAR % | 2.9 | [1.7, 4.1] | <0.001 |
| **T2DM patients (n=20):** |  |  |  |
| Δ Leptin% | 4.8 | [2.5, 7.1] | <0.001 |
| Δ Adiponectin % | -4.4 | [-7.8, -0.9] | 0.016 |
| Δ LAR % | 6.4 | [3.3, 9.5] | 0.001 |
| **Patients without DM (n=32):** |  |  |  |
| Δ Leptin% | 2.4 | [1.7, 3.2] | <0.001 |
| Δ Adiponectin % | 2.5 | [-4.5, 9.7] | 0.465 |
| Δ LAR % | 1.7 | [0.9, 2.4] | <0.001 |

**Supplementary Table 1:** **Relationship Between Percentage Change in Weight and Percentage Change in Leptin, Adiponectin and the LAR after Completion of the Milk-Based Meal Replacement Programme (adjusted).**

β denotes the beta coefficient and [confidence interval] for the estimate of the strength of the association between percentage weight change as the independent variable and relative changes in leptin, adiponectin and LAR as the dependent variables, adjusted for age, sex and baseline BMI, as well as the baseline measure of the dependent variable.
