# Supplementary material for: Changes in the Leptin to Adiponectin Ratio Are Proportional to Weight Loss After Meal Replacement in Adults With Severe Obesity
Source: Front Nutr. 2022 May 18;9:845574. doi: 10.3389/fnut.2022.845574 (PMC9158748; doi:10.3389/fnut.2022.845574)
Supplement: Supplementary file 2 [file Image_1.pdf]

Supplementary Figure 1 (a-c): Correlations Between Percentage Change in Weight and Percentage Change in Leptin (a), Adiponectin (b) and the LAR (c) after Completion of the Milk-Based LELD in Subgroup of Patients with T2DM.

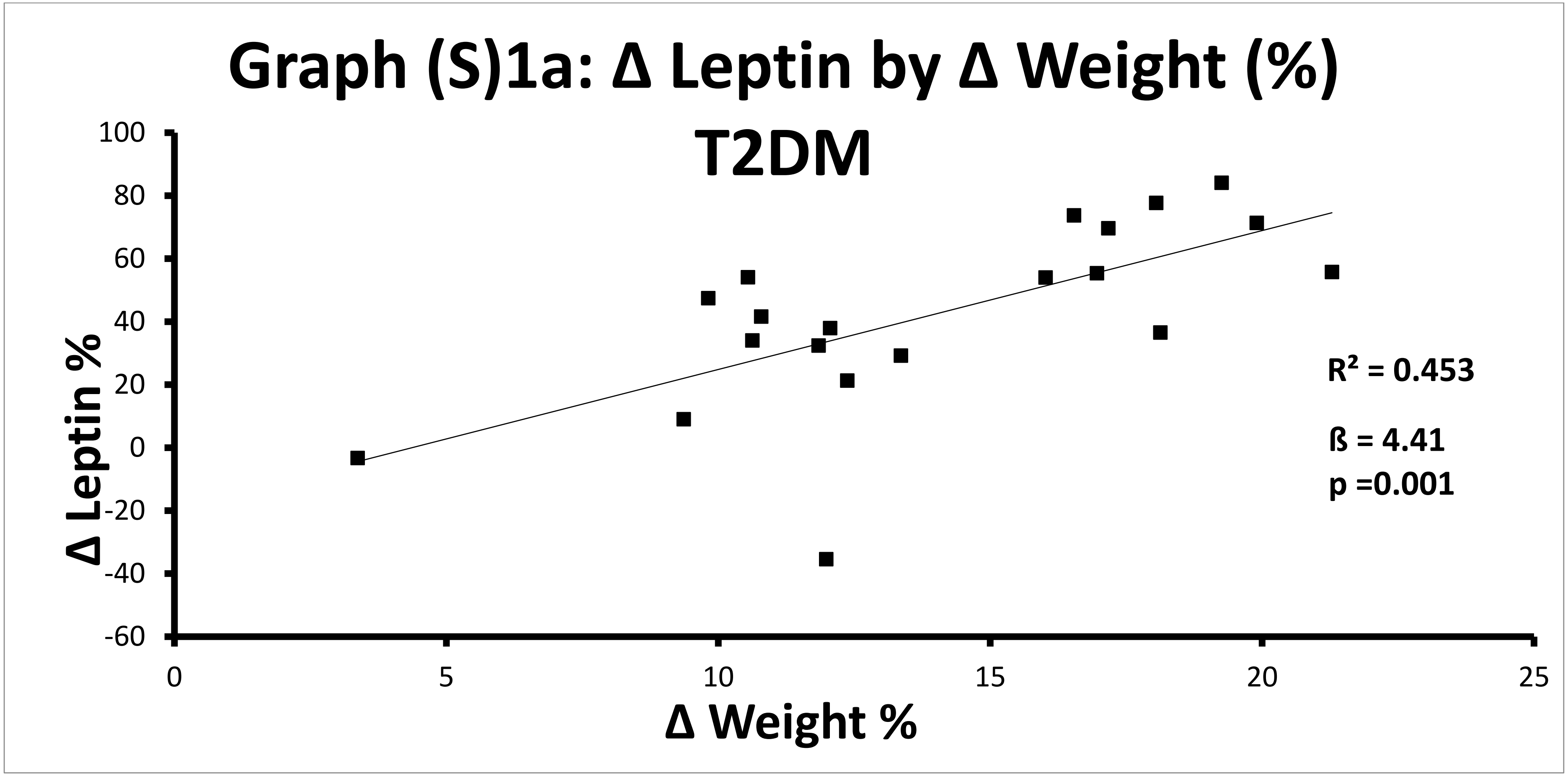

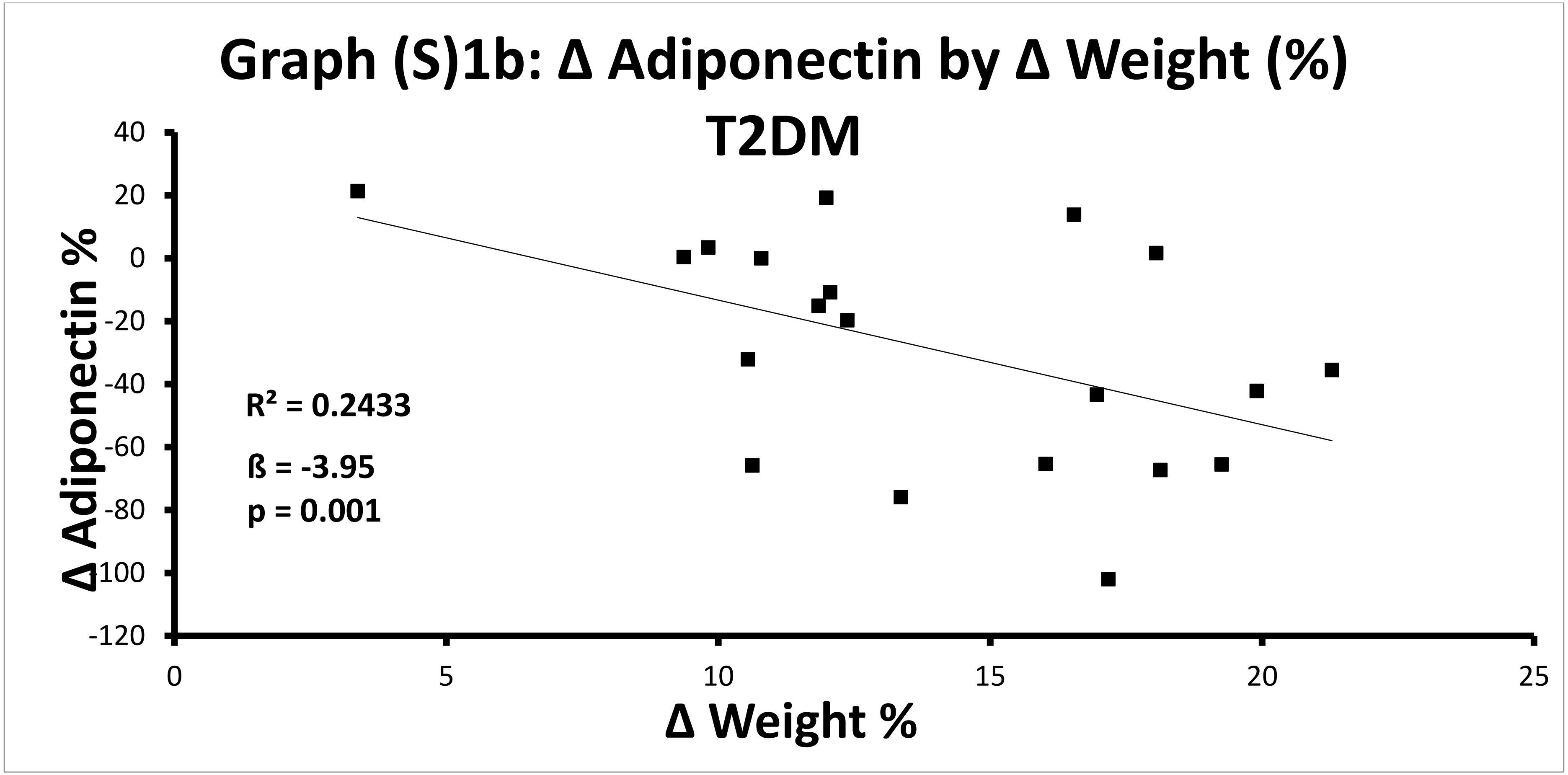
Graph (S)1b: Δ Adiponectin by Δ Weight (%)
T2DM
Δ Adiponectin %
Δ Weight %
R² = 0.2433
ß = -3.95
p = 0.001
40
20
0
-20
-40
-60
-80
-100
-120
0
5
10
15
20
25

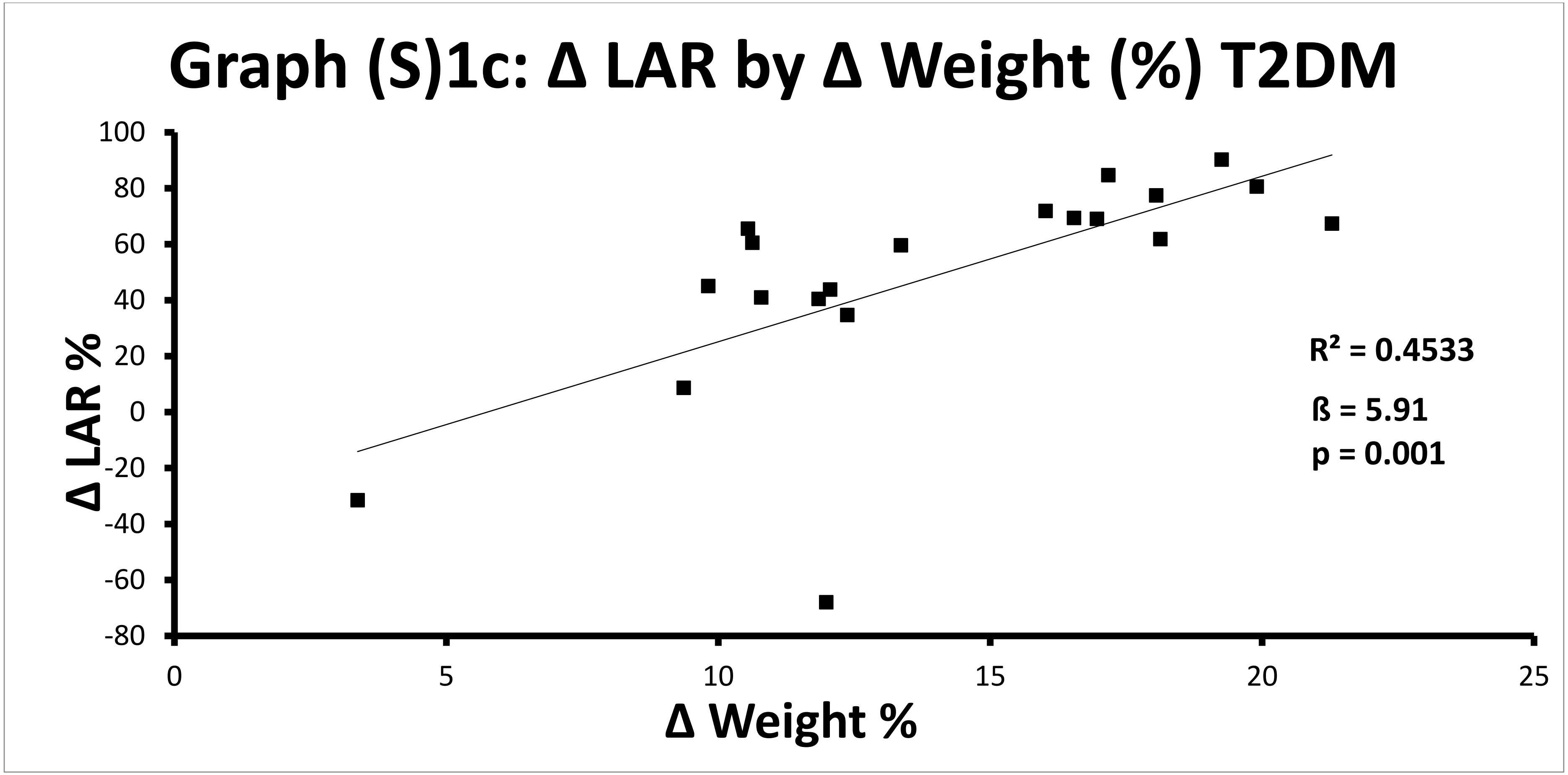

Graph (S)1c: Δ LAR by Δ Weight (%) T2DM
100
80
60
40
20
0
-20
-40
-60
-80
0
5
10
15
20
25
Δ LAR %
Δ Weight %
R² = 0.4533
ß = 5.91
p = 0.001
